# Biotechnological Approaches: Gene Overexpression, Gene Silencing, and Genome Editing to Control Fungal and Oomycete Diseases in Grapevine

**DOI:** 10.3390/ijms21165701

**Published:** 2020-08-09

**Authors:** Luca Capriotti, Elena Baraldi, Bruno Mezzetti, Cecilia Limera, Silvia Sabbadini

**Affiliations:** 1 Department of Agricultural, Food and Environmental Sciences, Marche Polytechnic University, 60131 Ancona, Italy; l.capriotti@pm.univpm.it (L.C.); b.mezzetti@staff.univpm.it (B.M.); cnlimera1983@hotmail.com (C.L.); 2 Department of Agricultural and Food Sciences, University of Bologna, Viale G. Fanin 42, 40127 Bologna, Italy; elena.baraldi@unibo.it

**Keywords:** plant biotechnology, *Vitis vinifera*, RNA interference, genome editing, biotic stresses

## Abstract

Downy mildew, powdery mildew, and grey mold are some of the phytopathological diseases causing economic losses in agricultural crops, including grapevine, worldwide. In the current scenario of increasing global warming, in which the massive use of agrochemicals should be limited, the management of fungal disease has become a challenge. The knowledge acquired on candidate resistant (R) genes having an active role in plant defense mechanisms has allowed numerous breeding programs to integrate these traits into selected cultivars, even though with some limits in the conservation of the proper qualitative characteristics of the original clones. Given their gene-specific mode of action, biotechnological techniques come to the aid of breeders, allowing them to generate simple and fast modifications in the host, without introducing other undesired genes. The availability of efficient gene transfer procedures in grapevine genotypes provide valid tools that support the application of new breeding techniques (NBTs). The expertise built up over the years has allowed the optimization of these techniques to overexpress genes that directly or indirectly limit fungal and oomycetes pathogens growth or silence plant susceptibility genes. Furthermore, the downregulation of pathogen genes which act as virulence effectors by exploiting the RNA interference mechanism, represents another biotechnological tool that increases plant defense. In this review, we summarize the most recent biotechnological strategies optimized and applied on *Vitis* species, aimed at reducing their susceptibility to the most harmful fungal and oomycetes diseases. The best strategy for combating pathogenic organisms is to exploit a holistic approach that fully integrates all these available tools.

## 1. Introduction

Grapevine is one of the world’s most commonly produced fruit crops, with a yield of about 79 million tons of grapes produced only in 2018 (Faostat Database, 2018). High-quality grapes for table consumption and wine production are derived from varieties of only one vine species, *Vitis vinifera* L. subsp. *sativa*, whereas other species are exploited as rootstocks [1], or are used in breeding programs solely for introducing new important traits in selected cultivars [2]. However, this kind of application is somewhat controversial due to international rules, particularly in Europe, where a limitation is imposed on the use of cultivars derived only from *Vitis vinifera* within breeding programs [3].

Considering their high pedoclimatic adaptation capacity, the cultivation of *Vitis vinifera* cultivars become possible between 30° to 50 °N and S latitude [4]. In the presence of favorable weather conditions (generally mild temperatures and high humidity), during the crop cycle, almost every organ of the plant is susceptible to attack by the main fungal and oomycetes diseases, such as downy mildew, powdery mildew, and grey mold, caused by *Plasmopara viticola* (Berk. and Curtis) Berl. and De Toni, *Erysiphe necator* Schwein., and *Botrytis cinerea* Pers., respectively [5]. According to recent global surveys, these diseases in regards to the main winegrowing regions in the world were considered by researchers and production professionals as the most harmful for grape production [6,7,8,9].

Grapevine breeding programs are mainly focused on inducing resistance against biotic agents, especially those that have a history of attacking European *Vitis* since the late nineteenth century, such as grape phylloxera and mildews. Mildews originated from North America, and they were introduced in most European *Vitis vinifera* varieties that proved to be highly susceptible due to the absence of coevolutionary processes between pathogens and plants [10]. Research work on resistant (R) genes and their introduction in selected cultivars for genetic improvement by classical breeding is generally challenging and requires several generations of backcrosses, during which a strict selection must be carried out, trying to synchronously preserve either important agronomic/oenological characteristics or traits of interest. The varietal rigidity imposed by registered designations of origin, long juvenile phase, and high heterozygosity leads to costly and longer breeding technical times when classical breeding is applied on *Vitis* species [11]. Furthermore, although classical breeding and agrochemical approaches were considered effective at first, in the long run, they could lead to the emergence of resistant pathogen strains, since they mainly confer a qualitative type of resistance which is prevalently monogenic [12]. To cope with these threats, farmers have massively used pesticides, arousing conflicting opinions regarding environmental sustainability and the quality of viticulture and wine production processes [13].

Studies in the field of plant molecular biology and biotechnology may support plant defense strategies, allowing researchers to select traits that could undermine the pathogen’s aggression [14]. Genetic transformation remains generally the most commonly exploited strategy compared to several other biotechnological approaches, as it allows researchers to stably insert specific gene sequences into a host plant. Genetic transformation also permits the importation of more than one R gene, and this creates the condition to have potential additive or synergistic effects. The validation of R genes in *Vitis* species is possible, but it requires the development of efficient regeneration and transformation protocols in order to genetically transform these plants, which often lead researchers to opt for model plants like *Nicotiana tabacum* or *Arabidopsis thaliana*, as hosts to implement these studies [15]. Classical genetic engineering techniques, mainly based on standard genetic transformation methods through the insertions and consequent expression of heterologous genes of interest, such as resistance (R) genes or other defense genes, represent the basis on which the new generation of biotechnologies are founded [11].

Overexpression of defense genes against crop fungal pathogens symbolizes one of the main biotechnological tools exploited to counterbalance pathogen aggressiveness, and consequent yield losses [14,16]. Pathogenesis-related proteins (PR proteins), antimicrobial peptides, secondary metabolites, and specific compounds can be overexpressed in host cells with a direct effect at the target level. Alternatively, it is also possible to stimulate host defense biosynthetic pathways (e.g., through the overexpression of transcription factors that enhance the plant defense-related genes) [17]. In addition to these biotechnological strategies, new breeding techniques (NBTs) such as genome editing mediated by CRISPR/Cas9 technology, a high precision tool capable of strategically introducing targeted mutations in the host genome [18], or cisgenesis/intragenesis which allow the inclusion of gene sequences from sexually compatible plants [19], have been developed and optimized during recent decades. The RNA interference (RNAi) mechanism, where double-strand RNA (dsRNA) molecules trigger the mRNA degradation or translational repression, is another powerful tool to subvert pathogenic attacks while the downregulation of gene expression occurs [20,21].

This literature review aims to provide an overview of target genes discovered in *Vitis* species and assessed through the abovementioned biotechnological strategies to increase tolerance to the most severe pathogens, such as grey mold, powdery and downy mildews.

## 2. Plant Response Mechanisms to Pathogenic Attacks

Plants have a series of biochemical or physical barriers that belong to the general constitutive defenses which can prevent fungi from entering the plant cells. Plant cell wall and related compounds such as trichomes, wax layers, cuticle, cellulose, and pectin lamellae are the main physical impediments to fungal ingression. For example, the presence of numerous trichomes on the lower leaf epidermis reduces downy mildew primary infection due to the increased exposure of zoospore to dehydration [22]. After infection, several histological responses help plant cells by curbing pathogen invasion. Callose deposition-forming cell wall thickenings, commonly known as papillae, lignin, and other phenolic compounds production nearby fungal penetration sites, have shown an active defense role during the early stages of plant invasion [23]. For instance, the expression of stress-induced callose synthase PMR4 (Powdery Mildew Resistance 4) has proven to provide complete penetration resistance against *Arabidopsis* powdery mildew [24].

During evolutionary-conserved plant defense processes, two effective and subsequent mechanisms occur to actively respond to pathogen and pest infections; firstly, the activation of the response machinery takes place following the perception of non-specific molecules produced by the pathogen; secondly, there occurs a specific recognition of pathogens virulence factors, through the products encoded by R genes [25]. Plants are able to generally recognize bacteria, fungi, oomycetes, and viruses thanks to either the presence or the production of specific conserved molecules, known as microbe- or pathogen-associated molecular patterns (MAMPs or PAMPs) that act as elicitors of plant defense responses [26]. In the case of pathogenic attack, the presence of some receptors and co-receptors known as pattern recognition receptors (PRRs) on the plant surface at the membrane level, can efficiently recognize PAMPs, thus, allowing the establishment of PAMP-triggered immunity (PTI) defense response, leading to the impediment of early-stage fungal growth, without killing the pathogen cells [27].

In order to suppress these barriers, pathogens can secrete a plethora of effectors which, in some cases, can be identified by the plant cell thanks to the presence of Resistance proteins (R proteins), which lead to the Effector-triggered Immunity (ETI) [28]. This plant immunity response is stronger than PTI, as it is able to elicit the activation of additional defense signaling mechanisms including PR genes expression induction, local hypersensitive responses (HR), and consequently, programmed cell death (PCD) [29]. Unfortunately, due to the lack of specific R genes, the most important *Vitis vinifera* cultivars have proved to have inadequate defense responses to limit the invasion of both biotrophic and necrotrophic Fungi and Chromista pathogens [5].

## 3. Genetic Engineering for the Expression of Candidate Genes Involved in Fungal-Oomycete Resistance

Prior to genetic transformation processes, a fundamental part is the identification of candidate genes that exert in the host an active role in the enhancement of plant defenses, such as pathogenesis-related proteins, antimicrobial peptides, transcriptional factors, products of the secondary metabolism, and defense-related genes. The expression/overexpression through genetic engineering techniques is still one of the most common biotechnological tools used to validate cisgenic and transgenic sequences that induce/improve resistance against specific pathogens in *Vitis* spp. (Table 1).

### 3.1. Overexpression of Pathogenesis-Related Proteins

**Figure 1:**
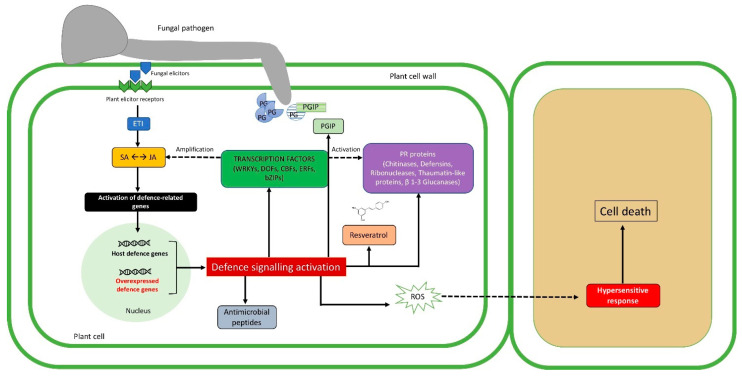
Description of transgenic plant–pathogen arms race during pathogenesis. The first impediment to fungal invasion is represented by chemical and physical barriers already existing before the infection. The trophic activity begins with lytic enzyme production (e.g., polygalacturonase, PG), that can be suppressed by the production of specific inhibitors (e.g., polygalacturonase inhibitor, PGIP), which can be expressed also by the use of genetic engineering techniques. A specific recognition takes place when elicitors coded by *avirulence* (*Avr*) genes of the fungal cell are recognized by host receptors, driving effector-triggered immunity (ETI). ETI results in the activation of defense gene expression (i.e., defense molecules, antimicrobial peptides, phytoalexins), through the salicylic/jasmonic acid (SA/JA) signaling pathways [28]. The aforementioned defense molecules together with pathogenesis-related proteins (PRs) and transcription factors can be overexpressed in the host cell, imparting a harmful effect against the pathogen. Intriguingly, transcription factors are responsible for activating plant defense response, and their overexpression leads to the stimulation of SA/JA signaling pathways. Solid arrows and dashed arrows indicate direct or indirect induction processes, respectively.

Once elicitors have been recognized by the plant, the contact with the pathogen induces different defense mechanisms in host cells, such as the reinforcement of structural barriers, the synthesis of secondary stress-related metabolites such as phytoalexins, and the provision of PR proteins [69], as depicted in Figure 1.

The extreme result of ETI is the hypersensitivity response that, thanks to reactive oxygen species (ROS) accumulation, leads to programmed cell death (PCD), isolating and detaining the propagation of the pathogen in other plant cells [29].

PR proteins (PRs) are a class of soluble proteins that were isolated for the first time from tobacco tissues after infection with the tobacco mosaic virus (TMV) [70]. In general, all the proteins expressed in response to both abiotic and biotic stresses are included in this category [17]. The expression of some of these proteins can also be triggered by the accumulation of plant hormones, such as salicylic acid (SA), jasmonic acid (JA) and ethylene (ET), which are related to plant defense [17]. PRs are relevant in resistant responses against fungal attack as they are generally involved in the formation of necrotic lesions that limit pathogen invasion and growth. Furthermore, they are activated in different ways depending on the pathogen trophic behavior. Biotrophs turn on the SA pathway in the plant, that triggers *NPR1* gene (*non-expressor of PR gene 1*) expression, which, in turn, induces the transcription and production of the SA-mediated gene proteins (*PR1*, *PR2*, *PR5*) that circulate in the sap, giving rise to Systemic Acquired Resistance (SAR) [17,71]. The overexpression of the *Vitis vinifera NPR1.1* gene increased resistance to powdery mildew in this species through the constitutive activation of *PR1* and *PR2* genes expression also in uninfected plants [30]. Necrotrophs induce the activation of the JA pathway in the plant, which induces the local accumulation of JA-mediated proteins (*PR4, PR5, PR12*), leading to Local Acquired Resistance (LAR) [17]. At present, 17 PR families have been classified from different plant species and some of them have shown evident antifungal activity such as β 1-3 glucanases (PR2) [72], chitinases (PR3, PR4, PR8, PR11) [73,74,75], thaumatin-like proteins (PR5) [73], proteinase inhibitors (PR6) [76], peroxidases (PR9) [77], ribonuclease like-proteins (PR10) [78], defensins (PR12) [79], and thionins (PR13) [80].

Chitinases and β 1-3 glucanases show antifungal activity thanks to their direct attack on the fungi cell wall, causing its fragmentation and disaggregation [34]. Two *Vitis vinifera* transgenic lines, expressing the *rice chitinase* gene (*RCC2*), exhibited HR and a significant reduction in powdery mildew symptoms (suppression of both conidial germination and mycelial growth) caused by *Erysiphe necator* and slight resistance to anthracnose when compared with the non-transformed control [31].

Bornhoff and collaborators showed the ineffectiveness of protection against powdery and downy mildew in transgenic *V. vinifera* plants expressing chitinase and RIP (Ribosome-inactivating protein) isolated from barley [32], contradicting the expectation of having synergistic effect due to the coexpression of these two enzymes. On the contrary, Nookaraju and co-authors observed a low susceptibility to *Plasmopara viticola*, characterized by a 15 to 35% reduction in hyphal growth in plants expressing both *chitinase* and *β 1-3 glucanase* genes [34]. By using the gene construct codifying for the *rice chitinase* gene *chi 11*, researchers obtained two grapevine transgenic lines characterized by a high chitinase activity, which displayed smaller lesions and delayed manifestation of powdery mildew symptoms [33]. Many chitin-binding proteins belong to the PR4 family, described mainly as wound-inducible proteins, and triggered by fungi infection in several plants. The isolation from *V. pseudoreticulata* of a PR4 protein and its overexpression in the susceptible *V. vinifera* genotype Redglobe led to an increased resistance to powdery mildew, inhibiting hyphal growth [35].

Thaumatin proteins belong to the PR5 family and are characterized by a thaumatin domain and a PR5-like protein kinase receptor [37]. Their anti-oomycete mechanism relies on their β 1-3 glucan binding and endoglucanase activities [38]. Dhekney and colleagues obtained two cisgenic grapevine lines showing broad-spectrum antifungal resistance, by expressing a gene construct codifying for the *Vitis vinifera* thaumatin-like protein 1 (VVTL-1), that conferred a 10 day delay in symptoms manifestation, compared to the non-transformed control after powdery mildew infection, and a significant resistance to black rot, a fungal disease caused by *Guignardia bidwellii* [36]. Transgenic *Vitis vinifera*, expressing a thaumatin-like protein gene isolated from *Vitis amurensis*, showed decreased susceptibility to downy mildew, reducing the infected area and the number of sporangia [38]. Further studies in this field suggested that some thaumatin-like protein (*TLP*) genes, perform better against biotrophs rather than necrotrophs, like *TLP29* gene of *V. quinquangularis* (*VqTLP29*) expressed in *A. thaliana*, where an increased susceptibility to *B. cinerea* was detected [37]. PR10 proteins are highly expressed after pathogen invasion, and their antifungal capability seems to also be associated with their RNase/DNase activity, and to their role in the control of flavonoid biosynthesis [81,82]. The *VpPR10.1* gene inserted through *Agrobacterium*-mediated transformation in Thompson Seedless cultivar led to reduced hyphal growth of *Plasmopara viticola*, through callose deposition around hyphae and haustoria, and hydrogen peroxide accumulation compared to non-transgenic lines [39].

### 3.2. Gene Expression of Antimicrobial Peptides

In addition to PR proteins, scientists revealed that some antimicrobial peptides (AMPs) were proved to have antifungal activity [83]. However, overexpression of AMPs does not always result in an enhanced resistance against fungi, maybe due to the activity of endogenous proteases which can inactivate peptides, neutralizing their antimicrobial properties [84]. Magainins are a class of antimicrobial peptides that interfere with fungal membranes function, altering their polarity and inducing cell mortality, but they do not interfere with the membrane of host cells. Grapevine plants overexpressing either natural or synthetic magainins have shown enhanced resistance to *Agrobacterium vitis* and *Erysiphe necator* [40]. In another study, the ectopic expression of *Magainin-2* (*mag 2)* and *PGL* genes generated plants slightly resistant to powdery mildew [41].

### 3.3. Overexpression of Transcriptional Factors

The role of transcriptional factors is strictly correlated with signaling pathways, and they play the role of regulating the expression of *PR* genes or enzymes implicated in defense responses [85]. The three more relevant transcription factor families that are unique in plants are WRKY proteins, ethylene-responsive element-binding factors (ERFs), and basic-domain leucine-zipper (bZIP) [86].

The WRKY proteins family includes 74 *Arabidopsis* members having the aminoacidic sequence WRKYGQK and a zinc finger-like motif in common [43]. Following *Plasmopara viticola* infection in grapes, *VvWRKY2* gene upregulation took place before the increased expression of *PR* genes, suggesting its involvement in phytopathogenesis [42]. In tobacco, the ectopic expression of the *VvWRKY2* gene led to a broad-spectrum resistance against fungi and oomycetes [42]. A reduction of up to 70% sporulation of *P. viticola* infecting grapevine leaves was also recorded in cisgenic plants expressing the *VvWRKY33* gene [43], and a 40% decreased susceptibility was recorded when 41B rootstock overexpressed the *VvWRKY1* gene [87].

In *Arabidopsis,* almost 124 ERF transcription factors are known to be involved in cold and drought tolerance as well as pathogen resistance [86]. The transcription factor VpWRKY11, that is a negative regulator of basal resistance in *Arabidopsis*, undergoes a proteolytic degradation operated by *Erysiphe necator*-induced RING finger protein 1 (EIRP1), thanks to its E3 ligase activity. Overexpression of the *VpEIRP1* gene in *Arabidopsis* showed enhanced resistance to powdery mildew and bacterial diseases [62]. The overexpression of three *ERF* genes isolated from *Vitis pseudoreticulata* showed a different level of tolerance against pathogenic fungi in tobacco and *Arabidopsis* plants, suggesting that more research is required for these transcription factors [45]. *Muscadinia* is another genus of *Vitaceae* family, characterized by plants naturally immune to different pathogens, including downy mildew. The overexpression of CBF2 transcription activator isolated from *M. rotundifolia* in *Arabidopsis* gave decreased susceptibility to downy mildew (*Peronospora parasitica*) and enhanced cold and drought tolerance, although it caused morphological changes and flowering delay [46].

Almost 75 transcription factors with a basic region/leucine zipper motif (bZIP) have been identified in *Arabidopsis*, which are able to regulate different mechanisms from plant defense signaling pathways to seed and flower development [88]. The ectopic expression of *bZIP60* gene, isolated from *Vitis vinifera*, decreased the severity of the symptoms of powdery mildew on *Arabidopsis* [47]. DOF transcription factors derived by the expression of the *DOF* gene family, which includes 25 members in *Vitis vinifera*, are involved in plant development and gene expression regulation [89]. The *VvDOF3* gene overexpressed in *Arabidopsis* acted as a transcription factor and increased resistance to powdery mildew [48].

### 3.4. Overexpression of Secondary Metabolites Generally Induced by Biotic Stress

Grapevine cultivation became also appealing from a nutraceutical point of view, due to the abundance of molecules with high antioxidant capacity contained in grapes. Stilbene, flavonols, and anthocyanins represent the major phenolic compounds that are responsible for generating beneficial effects also to plants, and they assist plant–pathogens arms races [90]. One of the most famous compounds, known for its anticancer property, is resveratrol, a stilbene polyphenol, constitutively present at low concentration in all grapevine organs. Resveratrol tends to accumulate in areas close to *Erysiphe necator*, *Plasmopara viticola,* and *Botrytis cinerea* infection sites, limiting pathogen spread and diffusion since these fungi are unable to metabolize this component. The antimicrobial and antifungal activity of resveratrol has been highlighted by some studies, showing that this compound displays an inhibitory effect on *Botrytis* conidia germination and mycelium growth, leading to ultra-structural changes on conidia (e.g., granulation of cytoplasm and disorganization of cell content) [91].

The biosynthetic pathway common to all the major phytoalexins in grapevine requires the activity of stilbene synthase (STS), which, by condensing three Malonyl CoA molecules with one molecule of Coumaryl CoA, leads to resveratrol production [51]. In vitro infection tests with *Botrytis cinerea* were carried out on grapevine micro cuttings transformed with the *V. vinifera Vst1* gene under the control of a promoter inducible by fungal infection (PR10 promoter isolated from alfalfa); the results obtained confirmed an enhanced resistance against the pathogen in the transgenic lines that exhibited the highest resveratrol synthesis level [51]. In another study, the expression of the *Vst1* gene under the cauliflower mosaic virus 35S promoter resulted in smaller botrytis necrotic lesion size in transgenic grapevine in comparison with control plants, demonstrating the existence of a positive correlation among resveratrol content and resistance to *Botrytis cinerea* [52]. In transgenic *Arabidopsis* plants, the expression of the *Vitis pseudoreticulata STS* gene (*VpSTS*), encoding a novel stilbene synthase, led to greater resistance to powdery mildew, since its expression seemed to be stimulated by SA, as happens with the expression of its homologous allele from susceptible *Vitis vinifera* [56]. The same *STS* gene was successfully introduced in Thompson Seedless somatic embryos, showing an increased concentration of resveratrol in the transgenic lines; however, no studies on increased pathogen resistance were carried out in this work [53].

On Chardonnay transformed plants, the same target genes led to an increased production of hydrogen peroxide and consequently, reduced mycelium growth of *Erysiphe necator* [55]. The *STS19* gene from Chinese wild grape was able to reduce powdery mildew and grey mold susceptibility when expressed in transgenic *Arabidopsis* plants, through SA/JA signaling pathways enhancement [57]. Although the genetic transformation aimed at improving the phytoalexin content seems to be a good strategy to reinforce plant immunity, at the same time, it can cause physiological and morphological alterations of the vine, especially during flowering and berry ripening [51].

Other molecular pathways are related to phytoalexin synthesis. It has been demonstrated that a positive correlation exists between calcium-mediated signaling and a high amount of resveratrol, corroborated by the fact that treatments with calcium channels blockers reduced resveratrol synthesis in transgenic *Vitis amurensis* cells [54]. The overexpression of the calcium sensor protein CDPK20 stimulated resveratrol production in *Vitis amurensis* cells, although only an increased *VaSTS7* gene expression was elicited, while the expression of other *STS* genes remained unaltered [54].

### 3.5. Overexpression or Gene Expression of Other Defense-Related Genes

Jasmonate Zim domain (JAZ) proteins are transcriptional repressors of JA signaling pathways, and participate in secondary metabolites biosynthesis, in addition to their involvement in response to biotic and abiotic stresses [59]. *VqJAZ4* gene (a jasmonate-ZIM gene from *Vitis quinquangularis*) transcription was induced after SA and MeJA application and by *Erysiphe necator* infection, evidencing its role in defense mechanisms [59]. Transgenic *Arabidopsis* plants expressing this gene showed enhanced resistance to powdery mildew and increased ROS accumulation and callose production, compared to non-transgenic control. However, the same *VqJAZ4* expressing lines appeared more susceptible to grey mold, possibly because *VqJAZ4* gene suppresses the expression of JA-related genes, and therefore, impeding JA signaling pathways activation, making the control of necrotrophs by the plant more thwarted [59].

One essential event for fungal and oomycetes pathogenesis is the production of polygalacturonases (PG) which acts in the impairment of host cell walls. The introduction of polygalacturonases inhibitors (PGIPs) in the plant through genetic engineering can limit the degradation of host cells and also as a result of enzymatic activity of cell walls compounds make available some glucosidic fragments that can react as effectors thus, eliciting host defense [60]. Twelve of the eighteen transgenic lines of *Vitis vinifera* expressing the pear *PGIP* gene showed a significant reduction in necrotic lesion size after inoculation with *Botrytis cinerea* [60]. In this study, the movement of PGIP through the xylem and the graft union was also demonstrated. This is an important result that shows the obtainment of genetically improved non-transgenic scion grafted on transgenic rootstock.

*Trichoderma* spp. are generally introduced in the field, notably in organic farming, as biocontrol agents for fungal disease management. The isolation of endochitinases and hexosaminidases from these biocontrol fungi and their genes transfer in grapevine cultivars allowed the obtainment of plants with enhanced tolerance to *Botrytis cinerea* and *Erysiphe necator* [92]. Ubiquitination in plants plays different strategic roles, especially regarding the selective degradation of proteins. The overexpression of the E3 ubiquitin ligases from *Vitis pseudoreticulata* led to an increased susceptibility in transgenic plants to powdery mildew [62].

Stimulation of HR represents a useful strategy, particularly effective for its rapid mode of action. It has been demonstrated that the metacaspases MC2 and MC5, identified in *V. rupestris* upon *Plasmopara viticola* inoculation, were involved in the execution of HR, activating the most efficient ETI in this genotype [29]. The ectopic expression of caspase-like regulators in plants related to cell apoptosis could increase resistance to broad-spectrum diseases, which mediates defense-related programmed cell death [29].

Melatonin is a molecule firstly discovered in mammals, where it acts as a fundamental regulator of circadian rhythms, and its presence was also confirmed in higher plants. Considering its innate antioxidant activity, this molecule can be used in agriculture both as a plant growth regulator and as a biostimulator to cope with stress conditions [93]. The melatonin biosynthetic pathway includes modifications of L-tryptophan and the acetylation of serotonin, operated by serotonin-N-acetyl transferase (SNAT). Expressing the *SNAT2* gene cloned from *V. vinifera* in *Arabidopsis* plants led to a greater accumulation of melatonin and reduced susceptibility to powdery mildew [64].

In the *Arabidopsis* genome, a locus that confers resistance to different mildews, *RPW8*, has been characterized and it is composed of two genes, *RPW8.1* and *RPW8.2* [94]; the latter is located in the extrahaustorial membrane that covers the fungal haustorium and promotes accumulation of hydrogen peroxide. The expression of this gene (characterized by an efficient inhibition of fungal growth and sporulation) in transgenic *Vitis vinifera* plants demonstrated that this resistance can be transferred in this species as well [65].

PTI in plants could be promoted by the expression of PRRs dedicated to PAMPs recognition. Transient expression of VaHAESA, a *V. amurensis* leucine-rich repeat receptor-like protein kinase, in grapevine leaves, determined significant reduction in an infected area by downy mildew, assisted by callose deposition, H_2_O_2_, and NO accumulation on the nearby infection site [68].

## 4. RNAi: Host- or Spray-Induced Gene Silencing against Fungi and Oomycetes

In addition to owning genetic heritage, nucleic acid molecules can be managed as tools that follow specific recognition by the vegetal cell and can be processed in various ways, giving a start to a set of signals that may stimulate the induction of different defense responses up to the block of specific mRNA translation [95].

Post-transcriptional gene silencing is arbitrated by the activity of small RNAs (sRNAs) [96]. In the plant kingdom, the class of sRNAs is represented by microRNAs (miRNAs) originating from endogenous *MIR* loci, and small interfering RNAs (siRNAs) derived from long double-stranded RNA (dsRNA) molecules [97]. Using RNA templates, RNA-dependent RNA polymerases (RDRP) are responsible for de novo synthesis of secondary siRNAs that exert a stronger role in gene silencing than primary siRNAs [98]. This eukaryotic conserved silencing mechanism allows transcripts degradation or protein production restraint, through sRNAs production having full or partial complementary sequences with the target mRNA. sRNAs are loaded into argonaute (AGO) protein, part of RISC complex (RNA-induced silencing complex), where they become probes for binding with complementary RNA targets, thus, exerting their silencing ability [20].

Host defense mechanisms and pathogen virulence strategies are linked through cross-kingdom mechanisms [96]. In the same way the plant sends siRNAs to silence target genes of the pathogens, the pathogen uses the same mechanism to increase its virulence by impairing host immunity genes [97,99]. Bidirectional cross-kingdom RNAi can be exploited for generating silencing effects through the introduction of RNA molecules in transgenic plants that can counteract fungal and oomycetes virulence genes [100]. Nowara and collaborators showed that the accumulation of dsRNAs in barley (*Hordeum vulgare*) and wheat (*Triticum aestivum*) targeting the fungal glucanosyl transferase, *Avrk1* and *Avra10* effectors caused a reduction in haustorium formation of the causative agent of powdery mildew *Blumeria graminis* [101]. Gene silencing of other effector proteins led to similar results against powdery mildew in *Hordeum vulgare* [102,103].

**Figure 2:**
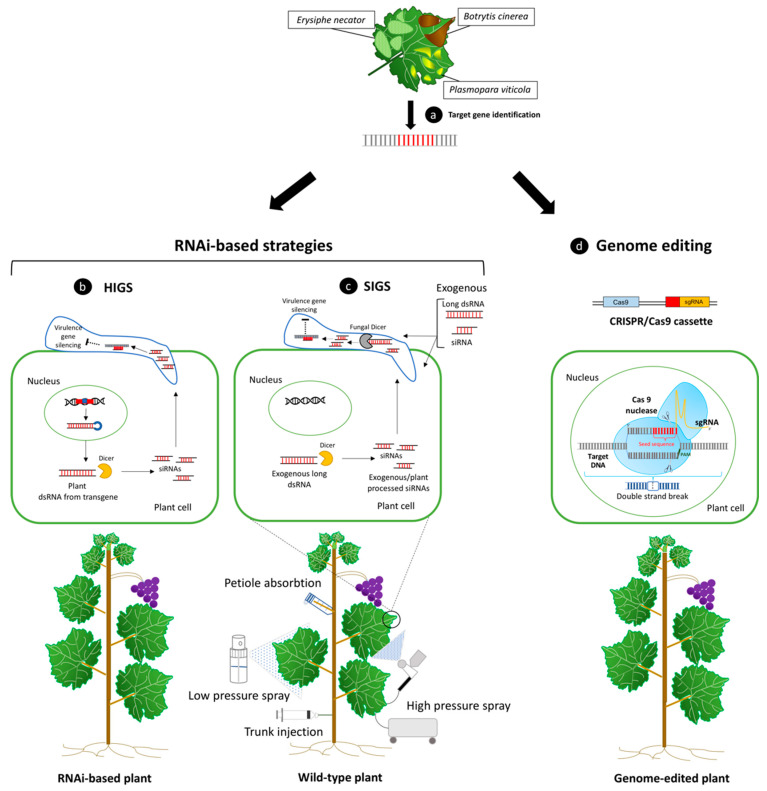
In addition to trans/cisgenesis methods, the expression of RNAi gene constructs in the plant, the exogenous applications of double strand RNA (dsRNA) molecules targeting host/pathogen genes, or plant genome editing, represent valid alternatives to enhance plant immunity during pathogenesis. (**a**) Candidate genes capable of limiting pathogen aggression or improve plant defense responses can be identified during the infection processes caused by the fungal and oomycetes causal agents of the most impactful diseases for grapevine production. RNAi-based strategies can be exploited to improve plant defense by providing dsRNAs to the plant cell through the expression of an introgressed hairpin-based gene construct in the plant genome, or through their delivery by exogenous application. (**b**) In host-induced gene silencing (HIGS), as a result of transcription of an RNAi sequence, a long dsRNA molecule is formed. When this molecule is recognized by Dicer-like protein, it is cleaved into siRNAs, which can knockdown related target gene expression [100]. (**c**) A transgenic-free procedure in which dsRNAs are directly sprayed on the surface of plants and pathogens is known as spray-induced gene silencing (SIGS). These molecules can be absorbed by both types of cells, and, depending on the delivery method used, dsRNAs can be processed by either the fungal/oomycetes and host RNAi machinery, leading to virulence gene knockdown and reduction in pathogen detrimental effects. Low-pressure spray, high-pressure spray, petiole adsorption, and trunk injection of dsRNAs represent some of the different available exogenous dsRNA delivery methods to confer plant protection against different plant pathogens, included fungi [143]. d) CRISPR/Cas9 system can be used for inducing targeted genome editing in plants, including the inactivation of specific plant susceptibility genes expression, which can help to regulate plant–pathogen interaction processes and disease resistance enhancement. Cas 9 protein complex is guided by artificially designed single guide RNA molecule (sgRNA) and leads to double-strand breaks (DSBs) of targeted DNA. SgRNA contains a seed sequence (around 8–12 bp, shown in red) complementary to target DNA that guides the binding of the Cas 9 protein to the target genomic sequence. The site of cleavage takes place three nucleotides upstream to the protospacer adjacent motif (PAM, shown in green) [20].

Once one or more genes to be silenced have been identified by studying plant–pathogen interaction processes (Figure 2a), several approaches can be used to deliver dsRNAs into plants. The potential of a host-induced gene silencing (HIGS) approach in crop disease management can be explored through the expression of RNAi constructs against various target genes in the host, as shown in Figure 2b. Resistance to *Fusarium graminaerum* was obtained by HIGS approach in *Arabidopsis* and barley plants, by targeting the fungal sterol 14α demethylase (CYP51) [104]. Other *Fusarium spp*. related diseases were successfully controlled by HIGS strategy in embryogenic cells of banana targeting two FOC proteins, whose roles are strongly related to fungal growth, development, and pathogenesis [105] or *chitinase* genes in *Triticum aestivum* [106]. Barley-stripe mosaic virus-induced RNAi worked efficiently also against *Puccinia tritici*, hitting three pathogenicity genes, and reducing leaf wheat rust [107]. Downregulation of the *Verticillium dahliae hygrophobins 1* (*VdH1*) gene, important for microsclerotia production, led to reduced wilt symptoms in cotton transformed plants [108].

RNAi machinery has been demonstrated to be also functional against oomycetes pathogens. Significant reduction in *Phytophtora spp.* load and disease progression was recorded in HIGS potato plants targeting the *Avr3a* effector, and the *G protein-β-subunit 1* (*PiGPB1*) gene of this pathogen species [109,110,111].

HIGS strategy has also been applied in lettuce plants to express silencing constructs targeting *Highly Abundant Message* (*HAM34)* or *Cellulose Synthase* (*CES1*) genes of *Bremia lactucae*, making these plants resistant to downy mildew [112].

All these studies have demonstrated the efficacy of this technique (HIGS) in specific genes downregulation, highlighting their high potential that can be managed in future grapevine genetic improvement programs aimed at increasing resistance to biotic stresses.

In addition to gene silencing against an external pathogen, is it also possible to exploit the RNAi mechanism to target endogenous genes, that have a negative influence in the pathosystem. An example is the case of *MLO* (*Mildews Locus O)* genes with transmembrane domain, considered as susceptibility genes (S-genes), that alter vesicle-associated and actin-dependent defense pathways [113]. Knockdown of the *VvMLO7* gene through constitutive expression of long non-coding dsRNA led to a significant reduction in powdery mildew disease severity in the transgenic grapevine cultivar Brachetto [114].

However, the application of the HIGS approach is limited by poor public acceptance and strict legislative rules applied to GMO cultivation, and also by the lack of efficient in vitro regeneration and genetic transformation protocols for several crops, and more often, for all the genotypes within the same species [115].

The fact that simple exogenous application of polynucleotides can affect mRNA levels of important virulence-related genes of pathogens/plants without modifying the host genome, opens new opportunities for the development of new scientific techniques and crop improvement strategies [116]. Extracellular-self DNA and RNA could be also applied to the plants in order to stimulate their immune response [97]. The attack of siRNA production machinery in the pathogen, through RNAi, has the potential to inhibit the pathogen virulence itself. Wang and collaborators demonstrated that *Botrytis cinerea DCL1/2*-long dsRNAs, targeting expression of dicer proteins essential for sRNA production, exogenously applied on the surface of detached leaves and fruits of different plant species, including grapes, can be efficiently taken up by the necrotrophic fungus, providing a relevant protection against grey mold [100]. Similar observations were made when spray applications of long non-coding dsRNA molecules, which target three genes required for the biosynthesis of *Fusarium graminearum* ergosterol, efficiently inhibited the fungal growth at the sprayed (local) as well as the non-sprayed (distal) parts of detached leaves, probably due to the basipetal and acropetal transportation along the vascular system of the silencing signal [117]. In many cases, researchers preferred to adopt a multitarget approach, by silencing simultaneously two or more target genes entailing in pathogenesis. White mold and grey mold symptoms can be significantly decreased in *Arabidopsis* and *Brassica napus* leaves, respectively, through foliar application of 20 different dsRNAs targeting various genes, evidencing the possibility to counteract closely related fungi while applying the same dsRNAs molecules on various crops [118]. The same conserved target gene among various fungi, such as β2-tubulin gene of *Fusarium asiaticum,* could be selected for RNAi, altering the damaging effects afforded by *Fusarium* spp., *Botrytis cinerea*, *Magnaporthe oryzae*, and *Colletotrichum truncatum* [119].

In addition to HIGS, the exogenous application of long dsRNAs, small dsRNAs, and hairpin RNAs has been recently studied and proposed as a new environment-friendly crop protection tool [120]. Spray-induced gene silencing (SIGS) allows the adsorption of dsRNA by either plant cells and tissues, where it can be processed from host RNAi machinery and/or then progressively conveyed on pathogen cells, or directly adsorbed and processed by the fungal cell driving gene silencing through their own RNAi machinery (Figure 2c) [117,121]. However, the exact mechanisms behind the uptake of exogenous dsRNAs and their use to activate RNAi machinery in the plant and/or pathogen cells is still unclear, and they seem to be affected by the method of exogenous application used combined with the absorption capacity of different plant organs [122,123,124,125].

The appearance of pathogens-resistant strains to fungicide can be counteracted using fungicides with different modes of action or with a combined application of dsRNAs. A reduction in *Fusarium asiaticum* pathogenicity and resistance to phenamacril, caused by a mutation in the *myosin-5-gene* (*Myo-5*), was recorded with the continuous application of the phenamacril and dsRNA-*Myo-5* as treatments on wheat speaklets [126]. Long or small dsRNAs could be supplied to plants via low-pressure or high-pressure spray, petiole adsorption, or trunk injection [98,100,117,118]. Through petiole adsorption and trunk injection methods, dsRNAs were shown to be limited to the apoplast and transported only along the xylem, without penetrating the plant cell. These results were demonstrated by Dalakouras and co-authors that applied both these techniques into *Vitis vinifera*, observing that the delivered hairpin-RNAs (hpRNAs) were systemically transported and detected in leaves, distant from the treated area, from one up to 10 days post-application, but no siRNAs deriving from DCL-processed hpRNAs were found [122]. Furthermore, when siRNAs were applied by petiole absorption also into GFP-expressing *N. bethamiana*, no silencing effect on GFP transcripts was observed [122]. Nevertheless, it seems possible by using these exogenous application techniques to directly reach fungal or oomycetes that normally colonize the apoplast and xylematic tissue where, after undergoing internal processing, dsRNAs can exert their biological activity [98]. On the other hand, high-pressure spray of siRNAs had the potential to ensure both local and systemic gene silencing on tobacco plants [127].

Selecting fungicide sites of action, Nerva and colleagues constructed a single long dsRNA molecule that exerted protection against grey mold in vitro on grapevine detached leaves and grapes at post-harvest, applied through the high-pressure spray and petiole adsorption. Despite different levels of protection being recorded among the dsRNAs delivery methods, interestingly, all the techniques that facilitate the provision of intact dsRNA to the fungus were assumed as effective [128].

Some authors have also reported the possibility to use SIGS to target endogenous genes in plants and downregulate their mRNA levels both locally and systemically [116,127,129]. To our knowledge, the data present in the literature are limited to the foliar application of dsRNA molecules to silence transgenes expressed in model plant species, like *Arabidopsis* and *N. benthamiana*; however, these results open new scenarios for the use of SIGS also to target endogenous gene sequences, like susceptibility genes in grapevine and other crops, to enhance plant defense responses.

## 5. Genome Editing

Genome editing is a powerful technique that facilitates the generation of multiple types of genome modifications, like insertion, deletion, or mutation, having various implications in genetic studies of animal and vegetal cells [20].

Among the three typologies of engineered nucleases that are at the base of genome editing techniques, Clustered regularly interspaced short palindromic repeats (CRISPR)/CRISPR-associated 9 (Cas9) (CRISPR/Cas9) is a lower cost, simpler, and faster system compared with the other enzymes such as zinc finger nuclease (ZFN) or transcription activator-like effector nuclease (TALEN) [130]. Specific modifications in DNA sequence could be driven by the CRISPR/Cas9 reprogrammed system, which needs the insertion of a well-designed single guide RNA (sgRNA) molecule into cells through different types of vectors (Figure 2d) [130]. SgRNAs are constituted by a single molecule of RNA composed of specific crRNA-trascrRNA (transactivating RNA) chimera sequence [20]. Mutation efficiency could be notably increased by designing multiple target sgRNAs for one target gene [131]. Ren and collaborators introduced a single plasmid containing specific sgRNA through *Agrobacterium*-mediated transformation into Chardonnay suspension cells to alter the biosynthetic pathway of tartaric acid [132]. *L-idonate dehydrogenase* (*idnDH*) gene was successfully mutated using CRISPR/Cas9 system, without recording off-target events and highlighting the importance of high GC content in sgRNA sequence in order to obtain high efficiency in genome modifications [132]. In fact, previous research works carried out in other plant species showed that sgRNAs designed to have a GC content above 50% led to a higher editing efficiency; this might have been due to the final binding capability of these molecules to their targets, which, in some species, genomes have high GC contents in specific regions [133,134]. *Vitis vinifera Phytoene Desaturases* (*VvPDS*) was efficiently knockout in cell masses of Neo Muscat [135], Chardonnay, and 41B rootstock by CRISPR/Cas9 binary vectors expressing a sgRNA with 65% GC content [136]. Notably, the first application of target genome editing (TGE) for increasing resistance against *B. cinerea* in grapevine was reported by Wang and colleagues [131]. The mutations of the *VvWRKY52* gene, induced by TGE in Thompson Seedless transgenic plants, led to a significant reduction in *B. cinerea* colonies, especially in biallelic grapevine mutant lines [131]. Mutation efficiency driven by the CRISPR/Cas9 system is widely dependent on different factors (technical methods, plant genotype, gene target, in vitro regeneration, and selective conditions) as already known for genetic transformation techniques, and others specifically for this approach such as the choice of Cas9 promoter and sgRNA sequence [137]. As an alternative to classical genetic transformation, a plasmid-mediated procedure that can lead to the generation of transgenic-free new varieties, which is based on direct delivery of CRISPR/Cas9 ribonucleoproteins (RNPs) generally into protoplasts, is available [138]. Although in vitro plant regeneration of protoplast can be applied to some herbaceous species, in recalcitrant woody fruit plants species, the development of this technique is hampered by many factors, and attempts on grapevine are relatively recent and need further studies [138,139].

A plasmid-free method to obtain genome-edited plants was elaborated by Malnoy and collaborators, in which CRISPR/Cas9 RNPs were directly inserted in protoplasts of grapevine cultivar Chardonnay and apple cultivar Golden delicious. Grapevine protoplasts were obtained from embryogenic calli, and the induction of site-directed mutation of the *Mildew Locus O-7* (*MLO7*) gene was demonstrated, however, the regeneration of new genome-edited plants was not reported [140]. Direct delivery of CRISPR/Cas9 RNPs was also described by Osakabe and colleagues, who detailed the advantages and critical steps in the obtainment of mutated *IdnDH* grapevine plants regenerating from protoplast or directly regenerated after classical *Agrobacterium-mediated* transformation [138]. In whichever manner, genome editing technology could be effectively applied for grapevine susceptibility gene knockout, which would be a beneficial plant defense strategy. This is the case of some *Vitis vinifera* cultivars, where susceptibility against downy and powdery mildews was decreased through the exploitation of the CRISPR/Cas9 system, by transforming embryogenic calluses in order to induce target mutagenesis of specific susceptibility genes [141]. In the future, targeted genome editing can be exploited to insert new genes or modify genes regulating plant–pathogen interaction at the expense of pathogens. Different to other NBTs that are based on the introduction of foreign DNA sequences in the host genome, TGE represents an innovative method that can induce specific modification in the existing genome limited to the introduction of single-point mutations [142].

## 6. Biosafety Considerations and Overview of Breeding Technologies Applied to Enhance Resistance against Fungal and Oomycetes Disease in Grapevine

Nowadays, various methods for grapevine genetic improvement are available, including both traditional breeding methods and new biotechnological approaches. The development and application of each of these strategies is often linked to several technical advantages and disadvantages; furthermore, they often give rise to new biosafety issues and public concerns (Table 2). A brief description of the new breeding techniques, compared with traditional breeding systems, referring to the possibility of increasing in grapevine resistance to fungal and oomycetes diseases, is reported below.

Traditional breeding: With the application of this technology, it must be accepted that new cultivars will be similar to the original clones but not the same, evidencing the importance of assisted tools such as Marker Assisted Selection (MAS) that, thanks to the possibility of using molecular markers, can be employed to detect genes of interest [144]. Cultivars suitability for the wine market must be tested and classified as a new type of wine. Generally, this is a long-term program, that requires deep knowledge of genetic resources, and the new clones corresponding to the original clones recognized internationally for particular wine brands can be identified with difficulty [145,146]. Moreover, if resistance to diseases is provided by the insertion of R genes from less susceptible genotypes, it will be easily overcome by the onset of new pathogen strains [145].

Transgenesis: this technique allows the overexpression of both homologous and heterologous genes, including antifungal proteins. Whenever regeneration/transformation protocols are available, this technology can confer stable resistance to diseases to any grapevine cultivars, mostly preserving the agronomic characteristics of the original clone. Great potential is given by the high availability of gene vectors and selectable marker genes. The presence of transgenes in the plant genome and its release into the environment make risk assessment and public acceptance more difficult [147].

Cisgenesis/Intragenesis: these technologies make it possible to introduce genes originally present in the same species or in sexually compatible ones into one genotype, through genetic transformation [148,149,150]. Compared to transgenesis, despite the availability of several grapevine resistance genes, it is more difficult to create full cisgenic gene constructs due to the lack of efficient cisgenic promoters and selectable markers, thus, making the selection of stably transformed plants more complicated [20] (Table 2).

Gene silencing—HIGS-RNAi technology: HIGS uses the same transgenesis approach but the inserted RNAi gene construct can be designed with high specificity and minimization of off-target effects [20]. It is applicable to downregulate/modulate the expression of plant endogenous genes and to target genes of grapevine pests and diseases. The expression of a new short RNAi sequence instead of new proteins facilitates risk assessment [151]. With the increasing knowledge of pests, fungi, and virus genomes, this technology offers an effective and flexible tool for introducing stable resistances in grapevine cultivars.

Gene silencing—SIGS-RNAi technology: This is not considered a transgenic approach because it is not based on recombinant DNA technology, and it involves the application of small RNA molecules, with a much higher target effect. The new products are regulated as new natural molecules and not as GMO. To ensure better delivery of dsRNAs, new formulates and production systems that will reduce production costs are under validation [21]. It has been demonstrated that the SIGS pathway is greatly independent of the canonical defense pathways, hence, conferring a “less expensive” and efficient immunity to cells, in comparison to an active pattern- or effector-triggered immunity (PTI/ETI) in progress, which is expensive in terms of cellular energy [117].

Genome editing—CRISP/Cas9: this is the most recent technology for inducing target mutations in grapevine. The potential of this technology applied to grapevine depends on the identification of specific susceptibility gene sequences to be modified in the grapevine genome. Some important results have already been identified; however, the results can be affected by the type of target gene [114,132,138,140]. Different studies have demonstrated the risk of this technology in inducing off-target effects, even though it has reduced risk on the environment and on the consumer [152]. The efficiency of this technology also depends on the methods used for the insertion of CRISPR/Cas9 protein needed for genome editing. In the case of genome editing induced by genetic transformation, the new plants are definitely GMO and regulated as such. The limited availability of efficient regeneration protocols from somatic tissue or protoplasts remains the main limiting factor in applying this technology for targeting mutation in different grapevine cultivars.

## 7. Conclusions

Plants and pathogenic fungi/oomycetes are living organisms and their interaction give rise to a series of interlocking events culminating in plants immunity deficiency or vulnerability. The establishment of a plant disease is a complex mechanism whose resolution can be achieved by the application of integrating defense strategies. Gene overexpression, gene silencing, and genome editing are mainly used for studying gene functions and can be efficiently exploited to control pathogenic diseases caused by the Fungi and Chromista kingdoms. All these biotechnological approaches could be exploited for decreasing both pathogens’ virulence and plant susceptibility to diseases. In contrast to traditional breeding methods, the application of biotechnological techniques allows the breeder to act specifically at the gene level, avoiding the introduction of undesirable genes in the new improved grapevine cultivar. A detailed study of candidate genes involved during the infection process is required in order to select the best target for protecting plants or counteracting pathogenicity and virulence gene expression.

Regeneration and transformation of recalcitrant *Vitis vinifera* cultivars remains to be the biggest challenge for the application of genetic engineering-related biotechnologies [115,132]. Once engineered plants have been obtained and before their commercialization, they must be subjected to strict regulation in order to guarantee the safety of their products towards the environment and on humans thereof. For this reason, researchers started to develop alternative strategies to classical biotechnological tools such as SIGS or cisgenesis and intragenesis, avoiding the introgression in the host genome of foreign genes and the use of antibiotic resistance genes as selectable markers [115].

Altogether, these approaches have the opportunity to offer preservation of plant health during the pathogenic challenge by providing a broad spectrum of defense mechanisms, ranging from an overproduction of various compounds to RNA-mediated silencing, passing through specific gene inactivation.

## Figures and Tables

**Table 1:** Representative attempts of genetic transformation applied in *Vitis* species to enhance resistance against the most harmful fungal and oomycetes pathogens.

Gene Name	Gene Source	Description	Biological Effect	Host	References
**Pathogenesis-Related Proteins**					
***Non-expressor of Pathogenesis Related 1* (** ***VvNPR1.1*** **)**	*Vitis vinifera*	Key signal in salicylic acid pathway and local basal resistance to biotrophs	Enhanced resistance to *Erysiphe necator*	*Vitis vinifera* cv. Chardonnay	[30]
***Rice chitinase* (** ***RCC2*** **)**	*Oryza sativa*	PR protein Class I Chitinase	Major resistance to *Erysiphe necator* and slight resistance to *Elsinoe ampelina*	*Vitis vinifera* cv. Neo Muscat	[31]
***Chitinase and ribosome-inactivating protein* (** ***RIP*** **)**	*Hordeum vulgare*	These genes encode for two antifungal proteins	Susceptibility to *Erysiphe necator* and *Plasmopara viticola* infection equal to that of the control	*Vitis vinifera* cv. Seyval blanc	[32]
***Rice Chitinase* (** ***Chi 11*** **)**	*Oryza sativa*	Pathogenesis-related protein	Late and reduced manifestation of *Erysiphe necator* symptoms	*Vitis vinifera* cv. Pusa Seedless	[33]
***Chitinase and β-1,3-glucanase***	Scab-infected Sumai 3 wheat	Pathogenesis-related proteins	Chitinase was more effective than glucanase in conferring tolerance to *Plasmopara viticola*	*Vitis vinifera* cv. Crimson Seedless	[34]
***VpPR4-1***	*Vitis pseudoreticulata*	PR4 proteins are considered chitin-binding proteins	Improved tolerance to *Erysiphe necator*	*Vitis vinifera* cv. Red Globe	[35]
***Thaumatin-like protein* (** ***Vvtl-1*** **)**	*Vitis vinifera* cv. Chardonnay	Pathogenesis-related protein 5	Increased resistance to *Erysiphe necator* and *Elsinoe ampelina*	*Vitis vinifera* cv. Thompson Seedless	[36]
***Thaumatin-like protein*** **(** ***VqTLP29*** **)**	*Vitis quinquangularis* cv. Shang-24	Pathogenesis-related protein 5	Increased resistance to powdery mildew, but decreased resistance to *Botrytis cinerea*	*Arabidopsis thaliana*	[37]
***Thaumatin-like protein* (** ***VaTLP*** **)**	*Vitis amurensis* Rupr. “Zuoshan-1”	PR5 proteins have endo-β-1,3-glucanase activity; binding β-1,3-glucan	Reinforced resistance to *Plasmopara viticola*	*Vitis vinifera* cv. Thompson Seedless	[38]
***VpPR10.1***	*Vitis pseudoreticulata*	PR10 have in vitro ribonuclease activity	Increased tolerance to *Plasmopara viticola*	*Vitis vinifera* cv. Thompson Seedless	[39]
**Antimicrobial Peptides (AMPs)**					
***Natural Magainin-2* (*Mag2*)/*synthetic derivate* (*MS199*) **	Magainin extracts from the skin of *Xenopus laevis* frog	Magainins with broad-spectrum in vitro antimicrobial activity against bacteria and fungi	Resistance to bacterial diseases such as crown gall diseases, minor susceptibility against *Erysiphe necator*	*Vitis vinifera* cv. Chardonnay	[40]
***Magainin-2* (** ***mag2*** **) +** ***PGL***	Magainin extracts from the skin of *Xenopus laevis* frog	AMP belonging to the Magainins family	PGL protein seems to inhibit *Botrytis cinerea* spore germination	*Vitis vinifera* cv. Chardonnay	[41]
**Transcription Factors**					
***VvWRKY2***	*Vitis vinifera* cv. Cabernet Sauvignon	WRKY protein isolated after *Plasmopara viticola* infection	Increased tolerance to *Botrytis cinerea* and broad-spectrum fungal resistance	*Nicotiana tabacum* cv. Xanthi	[42]
***VvWRKY33***	*Vitis vinifera*	WRKY protein	Enhanced resistance to *Plasmopara viticola*	*Vitis vinifera* cv. Shiraz	[43]
***VpWRKY3***	*Vitis pseudoreticulata* accession “Baihe-35-1”	WRKY protein isolated after *Erysiphe necator* infection	Improved tolerance to *Ralstonia solanacearum*	*Nicotiana tabacum* cv. NC89	[44]
***Ethylene response factors* (*VpERF2* and *VpERF3*)**	*Vitis pseudoreticulata*	Transcription factor isolated after *Erysiphe necator* infection	Enhanced resistance to *Ralstonia solanacearum* and *Phytophtora parasitica* var. *nicotianae* Tucker	*Nicotiana tabacum* cv. NC89	[45]
***C-repeat-binding factor dehydration-responsive element-binding factor 1C* (*MrCBF2*/*DREB1C*)**	*Muscadinia rotundifolia* “Noble”	Transcription factor isolated after *Plasmopara viticola* inoculation	Enhanced resistance to *Peronospora parasitica*	*Arabidopsis thaliana* “COL0”	[46]
***bZIP transcription factor* (** ***VvbZIP60*** **)**	*Vitis vinifera* cv. Jing Xiu	Transcription factor that activates the accumulation of salicylic acid and the expression of PR1 protein	Enhanced resistance to powdery mildew	*Arabidopsis thaliana*	[47]
***DOF protein* (*VvDOF3*)**	*Vitis vinifera*	Protein involved in plant growth, development, and plant defense	Enhanced resistance to powdery mildew	*Arabidopsis thaliana*	[48]
***Tify protein* (*VvTIFY9*)**	*Vitis vinifera*	Protein highly expressed in leaves. Play an active role in SA pathway	Increased resistance to powdery mildew	*Arabidopsis thaliana*	[49]
***C2H2-type zinc finger protein* (*VvZFP11*)**	*Vitis vinifera*	This protein expression is regulated by salicylic acid and methyl jasmonate	Enhanced resistance to powdery mildew	*Arabidopsis thaliana*	[50]
**Secondary stress-related metabolites**					
***PR10 promot*** ***e*** ***r- Stilbene synthase* (*Vst1*)**	*Vitis vinifera* cv. Optima	Stilbenes production	Decreased susceptibility to *Botrytis cinerea*	41B rootstock (*Vitis vinifera* cv. Chasselas x *Vitis berlandieri*)	[51]
***Stilbene synthase* (*Vst1*)**	*Vitis vinifera*	Stilbenes production	Reinforced resistant against *Botrytis cinerea*	*Vitis vinifera* cv. Sugraone	[52]
***Stilbene synthase* (*STS*)**	*Vitis pseudoreticulata*	Stilbenes synthesis	Transgenic plants with high resveratrol content	*Vitis vinifera* cv. Thompson Seedless	[53]
***Calcium-dependent protein kinase* (*CDPK*) (*VaCPK20*)**	*Vitis amurensis*	Regulator of the biosynthetic pathways of resveratrol	Increased expression of *STS7* gene, enhanced resveratrol production	Cell cultures of *Vitis amurensis* rupr.	[54]
***Stilbene synthase* (*VpSTSgDNA2*)**	*Vitis pseudoreticulata*	Stilbenes production	Improved tolerance against *Erysiphe necator*	*Vitis vinifera* cv. Chardonnay	[55]
***Stilbene synthase* (*VpSTS*)**	*Vitis pseudoreticulata*	Stilbenes production	Improved resistance to powdery mildew	*Arabidopsis thaliana*	[56]
***Stilbene synthase* (*VaSTS19*)**	*Vitis amurensis*	Stilbenes production	Improved resistance to *Botrytis cinerea* and powdery mildew	*Arabidopsis thaliana*	[57]
***Stilbene synthase* (*VqSTS6*)**	*Vitis quinquangularis*	Stilbenoids accumulation	Improved resistance to *Erysiphe necator*	*Vitis vinifera* cv. Thompson Seedless	[58]
**Defense-related genes**					
***Jasmonate-ZIM domain protein* (*VqJAZ4*)**	*Vitis quinquangularis* clone Shang-24	This gene is upregulated after *Erisiphe necator* inoculation	Improved resistance to powdery mildew and enhanced susceptibility to *Botrytis cinerea*.	*Arabidopsis thaliana*	[59]
***Polygalacturonase-inhibiting proteins* (** ***pPGIPs*** **)**	Pear fruit	PGIPs are plant cell wall proteins that specifically inhibit fungal endo-polygalacturonases (PGs).	Increased resistance to *Botrytis cinerea* and slight tolerance to *Xylella fastidiosa*	*Vitis vinifera* cv. Thompson Seedless and Chardonnay	[60]
***Two endochitinases* (*ech42 and ech33*) *and one N-acetyl-*** ***β*** ***-d-hexosaminidase* (*nag70*)**	*Trichoderma harzianum, Trichoderma virens*	Extracellular endochitinases of biocontrol agents and chitinolytic genes	Enhanced resistance to *Botrytis cinerea.* Tolerance to *Erysiphe necator* in *ech*42-*nag*70 expressing transgenic plants	*Vitis vinifera* cv. Thompson Seedless	[61]
***E3 ubiquitin ligase Erysiphe necator-induced RING finger protein 1* (*VpEIRP1*)**	*Vitis pseudoreticulata* Baihe 31-1 accession	This protein activates plant defense response through the proteolysis of VpWRKY11 transcription factor	Enhanced resistance to powdery mildew	*Arabidopsis thaliana*	[62]
***F-box/Kelch-repeat protein* (*VpEIFP1*)**	*Vitis pseudoreticulata*	Transcription of EIFP protein is induced after powdery mildew infection and activation of PR genes	Enhanced tolerance to *Erysiphe necator*	*Vitis vinifera* cv. Red Globe and *Arabidopsis thaliana*	[63]
***Metacaspases* (** ***VrMC2* and** ***VrMC5*** **)**	*Vitis rupestris*	Executors of hypersensitive response (HR), isolated after *Plasmopara viticola* infection	Programmed cell death (PCD) activation	*Nicotiana tabacum* cv. Bright Yellow 2 and *Vitis vinifera* cell cultures	[29]
***Serotonin N-acetyltransferase* (** ***VvSNAT2*** **)**	*Vitis vinifera*	Protein essential for melatonin production and for SA and JA signaling pathways activation	Improved resistance to powdery mildew	*Arabidopsis thaliana*	[64]
***Resistance to Powdery Mildew 8 locus* (*RPW8.2*)**	*Arabidopsis thaliana*	Protein that encodes for small basic protein, with weak homology with NB-LRR protein	*Erysiphe necator* hyphal growth and sporulation were significantly restricted	*Vitis vinifera* cv. Thompson Seedless	[65]
***Ubiquitin ligase* (** ***VpPUB23*** **)**	*Vitis pseudoreticulata*	Type E3 ubiquitin ligase is involved in many immune regulation responses	Decreased resistance to *Erysiphe necator*	*Vitis vinifera* cv. Thompson Seedless	[66]
***Ubiquitin ligase* (** ***VaPUB*** **)**	*Vitis amurensis*	U-box protein E3 ligase causes downregulation of PR10	Transgenic plants were susceptible as control to *Plasmopara viticola*	*Vitis vinifera* cv. Thompson Seedless	[67]
***VaHAESA***	*Vitis amurensis* cv. Shuanghong	Pattern recognition receptor (PRR) that belongs to leucine-rich repeat receptor-like protein kinase	Induce H_2_O_2_, NO, and callose accumulation. Leaves showed less spores and *Plasmopara viticola* infected areas than control	*Vitis vinifera* cv. Thompson Seedless	[68]

**Table 2:** Description of different biotechnological approaches highlighting biosafety concerns and consumer acceptance.

Technology	Type of Modification	Target Origin and Description	Time Needed	Classification According to EU-Legislation	Side Effects	Biosafety Concerns	Consumer Acceptance (Proposed)
***Traditional breeding***	Breeding and several backcrosses generation, introgression breeding, induced mutagenesis, and somatic hybridization	Genes found in crossable, sexually compatible organisms	At least 10-15 years	Non-GMO	Altered clone identity, partial resistance to biotic stresses	No biosafety concerns and basic regulation needed [149]	High [153]
***Transgenesis***	Genetic transformation	Overexpression of genes also from non-sexually compatible organisms, presence of gene sequences (i.e., promoter, selectable marker gene) from non-compatible organisms	Around 1 or 2 years	GMO	Release in the environment of genes of different origins; expression of new protein products with possible allergen/toxic effects	Expression of unknown protein/enzyme; use of antibiotic/herbicide resistance markers, lack of coexistence with non-GM, organic cultivations	Low [147,154,155,156,157]
***Cisgenesis/Intragenesis***	Genetic transformation	Expression/overexpression of a gene originating from the recipient plant itself (cisgenesis), or expression of full/partial coding sequence originating from a sexually compatible plant (intragenesis)	Around 1 or 2 years	GMO	Scarce availability of efficient cisgenic selectable marker genes	cisgenic/intragenic plants solve the current biosafety problems regarding the presence of foreign genes in the plant host genome [158]	Medium/High [148,149,159]
***Gene silencing-HIGS***	Genetic transformation	Overexpression of non-coding dsRNAs downregulating exogenous or endogenous gene expression	Around 1 or 2 years	GMO	Efficacy of gene silencing varies with the genes and target organisms. Possible off-target effects in non-target organisms (NTOs)	Reduced off-target effects by designing RNAi sequences with high specificity and verified with bioinformatic studies. Minimal biosafety concerns when HIGS is applied only to rootstocks by trans-grafting technique [160]	Medium [161,162,163]
***Gene silencing-SIGS***	No genetic modification	External application of non-coding dsRNAs downregulating exogenous or endogenous gene expression	Few months	Non-GMO	Efficacy of gene silencing depends on the efficiency and specificity of the RNAi sequence and on the degree of adsorption showed by plants and pathogens cells	RNAi sequence should be selected in order to avoid off-target effects [143]. The absence of negative effects, that can be caused by the nanotechnology-based delivery method used, on the environment and human health needs to be demonstrated	Medium/High [21,164]
***Genome editing***	Genetic transformation/plasmid-free protoplast transformation	Artificially gene modification/target random mutation	About 1 or 2 years. More time necessary if transgene segregation is required from T0 plants, or if protoplast in vitro regeneration is required	Non-GMO/GMO in Europe (ECJ-2018)	Possible appearance of off-target mutations; difficulties in plant regeneration from protoplasts	Transgene integration, effect of the expression of Cas9 protein, specificity and fidelity of Cas9 protein [165]	Medium/High [166,167]
